# Soluble Interleukin-2 Receptor/White Blood Cell Ratio Reflects Granulomatous Disease Progression in Common Variable Immune Deficiency

**DOI:** 10.1007/s10875-023-01560-1

**Published:** 2023-08-05

**Authors:** Astrid C. van Stigt, Virgil A. S. H. Dalm, Nicole M. A. Nagtzaam, P. Martin van Hagen, Willem A. Dik, Hanna IJspeert

**Affiliations:** 1 Erasmus MC, University Medical Center Rotterdam, Laboratory Medical Immunology, Department of Immunology, Rotterdam, The Netherlands; 2 Erasmus MC, University Medical Center Rotterdam, Department of Internal Medicine, Division of Allergy & Clinical Immunology, Rotterdam, The Netherlands; 3 Erasmus MC, University Medical Center Rotterdam, Academic Center for Rare Immunological Diseases (RIDC), Rotterdam, The Netherlands

To the editor,

Granulomatous disease occurs in at least 8–20% of patients with common variable immune deficiency (CVID) and is associated with severe morbidity and mortality. Identification of CVID patients with granulomatous disease is crucial to prevent irreversible organ damage. However, detection and monitoring of progressive granulomatous disease in CVID remain difficult, most likely due to an insufficient understanding of the pathophysiology, the heterogeneous clinical presentation, and limited detection methods for in vivo small granulomas.

Granulomas can occur in any organ but mainly affect the lung. In CVID patients, the term granulomatous interstitial lung disease (GLILD) is used to describe interstitial lung disease with lymphocytic infiltrates with or without granulomas. A number of studies investigated potential biomarkers for GLILD in CVID, recently evaluated by Bintalib et al. [1]. GLILD has also been correlated well with splenomegaly, episodes of hematologic autoimmune diseases, low serum IgA levels, and an increased percentage of CD21^low^ B cells [2, 3]. Maglione et al. reported increased IgM in progressive CVID + GLILD [4]. Also, increased serum levels of sCD28, sCD83, IL-10, and LAMP3 were described in patients with GLILD [5]. Recently, Fraz et al. described that CVID patients with GLILD had elevated serum sIL-2R, sTIM-3, IFN-γ, and TNF-α, suggesting that T-cell activation, T-cell exhaustion, and possibly excessive macrophage activity are pathophysiological markers in CVID patients with GLILD [6]. A case report by Vitale et al. described increased IL-12 and sIL-2R as potential biomarkers for GLILD progression and treatment response, indicating sIL-2R increased in several inflammatory conditions [7]. Smits et al. showed sIL-2R, together with neopterin and IgM, declining significantly in CVID + GLILD patients after corticosteroid treatment and higher sIL-2R serum levels in more severe GLILD [8]. In our CVID cohort, we also found that sIL-2R correlated very well with granulomatous disease [9].

Having biomarkers for granulomatous disease or GLILD that can easily be implemented in routine diagnostics is important for risk stratification of CVID patients and could potentially be used as an indication to start or intensify immune suppressive treatment. However, most studies have not explored whether these biomarkers correlate with progressive granulomatous disease. While progressive granulomatous disease results in increased clinical symptoms and can lead to tissue damage, there is a need for intensified monitoring via CT scans and lung function tests and might require additional or intensified immune modulatory therapies. 

Previously, we showed that serum sIL-2R (also known as sCD25) increased during the progression of granulomas and declined upon treatment [9]. However, sIL-2R levels could not be used to discriminate between CVID with granulomatous disease and granulomatous disease progression. Therefore, we investigated if we could find a biomarker or combination of biomarkers that were previously described for detecting granulomatous disease progression in CVID. We performed a single-center retrospective cohort study comparing sIL-2R with serum levels of ACE, sCD163, sCD206, and sCD14 as markers of monocyte/macrophage activation, absolute cell counts of the white blood cells (WBC), T cells, CD4^+^T cells, CD8^+^ T cells, B cells, and NK cells. To determine whether these markers were different at the time of granuloma progression we made the following groups: (1) patients with non-progressive granulomatous disease (CVID + G, *n* = 7), (2) patients with progressive granulomatous disease (CVID + PG, *n* = 7), and (3) patients having infectious complications only (CVID IO, *n* = 23). Also, healthy controls (HCs, *n* = 11) and a group of untreated sarcoidosis patients (SARC, *n* = 18) were included, since misdiagnosis between granulomatous CVID and sarcoidosis may occur due to the similarities in clinical presentation (Table S1 and S2).

As we observed previously, sIL-2R is significantly increased in CVID + PG compared to CVID IO, HCs, and SARC and to CVID + G non-significantly (Fig. 1A) [9]. The levels of ACE and sCD163 were also significantly increased in CVID + PG compared to CVID IO and HCs, but they could not differentiate between CVID + G and CVID + PG or SARC. sCD206 was increased in CVID + PG versus HCs and SARC, but this was non-significantly compared to the other groups (Fig. 1A). In contrast to the study of Fraz et al., sCD14 was not increased in CVID + G nor CVID + PG, only in SARC compared to HCs (Fig. 1A). In line with Fraz et al., the absolute number of T cells (mainly CD4^+^T cells), B cells, and NK cells was lower in CVID + G, but especially in CVID + PG, compared to CVID IO and HCs, however not significant (Fig. 1B). The CD4/CD8 ratio was low in all CVID patients but high in patients with sarcoidosis. Interestingly, WBC count was low in CVID + PG. It was significantly reduced compared to CVID IO and non-significantly reduced compared to CVID + G and SARC (Fig. 1B). Since only three of the seven patients received immunomodulatory treatment at + PG, this unlikely explains the reduction in the WBC count (Supplemental Table 3). This decrease in WBC between + G and + PG was not specific for one cell type since both monocytes, neutrophils, and lymphocytes decreased at the + PG time point (Supp. Fig S3).

**Fig. 1:**
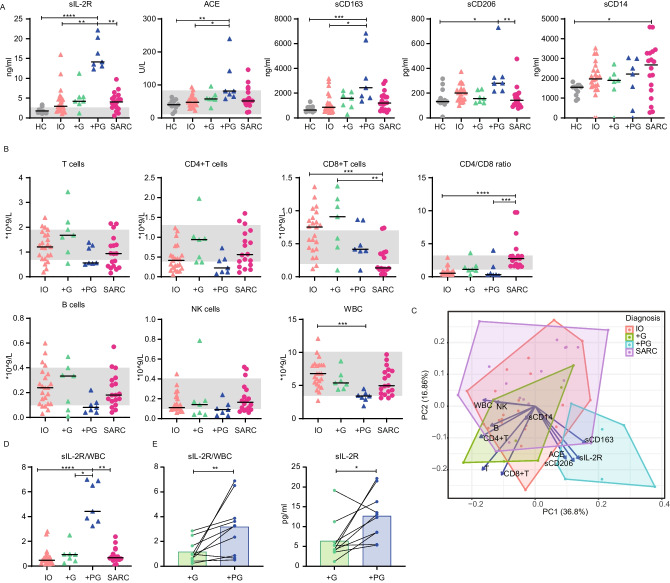
Soluble and cellular biomarkers for granulomatous disease in CVID. **a** Soluble serum markers measured in serum of healthy controls (HCs) (*n* = 11), CVID with infections only (IO) (*n* = 23), CVID with granulomatous disease (+ G) (*n* = 7), CVID with progressive granulomatous disease (+ PG) (*n* = 7), and sarcoidosis (SARC) (*n* = 18). **b** Absolute cell counts and ratios obtained for IO, + G, + PG, and SARC. Gray areas indicate the clinical reference values of the parameter applicable. **c** PCA of the four patient groups, including all serum soluble markers and cell counts using Log10 transformed data. Arrows indicate loadings of the parameters (**d**) the sIL-2R/WBC ratio of the four patient groups. **d** Paired analysis of 10 CVID patients with granulomatous disease, comparing + G versus + PG. Statistical analysis was performed with the Kruskal–Wallis test with the Dunn post hoc correction. One asterisk denotes *P* ≤ 0.05, two asterisks denote *P* ≤ 0.01, three asterisk denote *P* ≤ 0.0001

To determine whether a combination of all the markers could give a better differentiation between the groups, we performed non-supervised hierarchical clustering (Fig. S1). All CVID + PG patients were located in cluster 3, while the other groups were mainly in clusters 1 and 2. Principal component analysis (PCA) showed the majority of CVID + PG clustered separately from the other groups. The main drivers for clustering were sIL-2R, sCD163, sCD206, total WBC, and CD4^+^ T cells (Fig. 1C). The observed contribution of sCD163 and sCD206 suggests the importance of macrophage activation during granuloma progression, together with activated CD4^+^T cells. Interestingly, CVID + G showed more overlap with sarcoidosis and CVID IO than CVID + PG.

We searched for easy-to-implement biomarkers suitable for detecting granulomatous disease and disease progression in CVID. Although several of these markers fulfilled these criteria, measuring a large number of markers is too costly and complex. Therefore, we determined which markers we minimally needed. Since sIL-2R was the most discriminative soluble marker, and the WBC was strongly decreased in CVID + PG, we calculated the ratio of sIL-2R/WBC. This ratio was significantly higher in CVID + PG compared to all other groups (Fig. 1D). Receiver operator characteristics (ROC) analysis showed very high sensitivity and specificity which was comparable to sIL-2R alone (Fig. S2, Supplemental Table 3).

To further explore the association between sIL-2R and sIL-2R/WBC ratio with granuloma progression in CVID, we performed paired analysis at the time of non-progressive and progressive granulomatous disease (Fig. 1E). Therefore, we retrieved additional measurements for 10 of the 12 CVID patients with granulomatous disease. This revealed that granuloma progression was associated with an increase in the sIL-2R/WBC ratio and sIL-2R levels in 9/10 patients; however, the level of significance was slightly higher for the sIL-2R/WBC than sIL-2R (Fig. 1E). Although the increase in sIL-2R/WBC ratio and sIL-2R alone is very comparable, in some patients, the sIL-2R/WBC ratio was more indicative for progression of granulomas compared to sIL-2R alone.

In patient 6, episodes of collagen colitis complicated the clinical picture. At the time of CVID + G, the sIL-2R level was increased (5.3 ng/ml) compared to the reference range (4.2 ng/ml) and CVID IO (2.9 ng/ml). However, this high sIL-2R could be caused by the collagen colitis that evolved in the preceding year, since increased sIL-2R has been associated with inflammatory bowel disease [10]. The sIL-2R/WBC ratio was 0.9, which is in the range of CVID IO (0.2–2.8). In addition, patient 1 was diagnosed with GLILD and developed non-regenerative nodular hypoplasia suggestive of additional granulomatous lesions over time. When progression of GLILD and liver lesions were observed, the sIL-2R showed a 2.0-fold increase (4.2 to 8.5 ng/ml), whereas sIL-2R/WBC showed a 5.5-fold increase (0.6 to 3.3). Possibly, sIL-2R/WBC corresponded more accurately with the clinical context than the relative, moderate increase observed in sIL-2R.

So, although larger cohort studies are necessary to confirm our findings, we believe that the sIL-2R/WBC ratio is a more clinically accurate and discriminative monitoring tool for detecting and monitoring granulomatous disease and progression in CVID, as compared to sIL-2R alone or ACE. Both sIL-2R and the WBC count are cheap, easy-to-implement diagnostic measurements that mostly likely are already available in most routine diagnostic laboratories.

### Supplementary Information

Below is the link to the electronic supplementary material.

Supplementary file1 (PDF 911 KB)

## Data Availability

The datasets generated and analyzed during the current study are available from the corresponding author on reasonable request.
