# Supplementary material for: Soluble Interleukin-2 Receptor/White Blood Cell Ratio Reflects Granulomatous Disease Progression in Common Variable Immune Deficiency
Source: J Clin Immunol. 2023 Aug 5;43(8):1754–7. doi: 10.1007/s10875-023-01560-1 (PMC10661782; doi:10.1007/s10875-023-01560-1)
Supplement: Supplementary file 1 — Supplementary file1 (PDF 911 KB) [file 10875_2023_1560_MOESM1_ESM.pdf]

Supplemental data soluble interleukin-2 receptor/white blood cell ratio reflects granulomatous disease progression in common variable immune deficiency

Contents

Material & methods ... 2

Inclusion CVID patients and healthy controls ... 2

Categorization of CVID patients ... 2

Cytokine serum measurements ... 2

Absolute cell numbers collection ... 3

Analysis and statistics ... 3

Supplemental Table S1. Overview included participants, serum markers and cell counts as shown in Figure 1A-D ... 4

Supplemental Table 2. Additional characteristics and analysis groups CVID patients with granulomatous disease ... 6

Supplemental Table 3. ROC paramters and Youden's indek sIL-2R/WBC ratio and sIL-2R for groups as shown in Figure 1A-D ... 8

References supplementary data ... 9

Supplemental figures ... 10

Legends supplemental figures ... 12

Legend Supplemental Figure S1 ... 12

Legend Supplemental Figure S2 ... 12

Legend Supplemental Figure S3 ... 12

Material & methods

Inclusion CVID patients and healthy controls

Sera were obtained from 37 CVID patients (median 40, range 14-84 years, male n = 12, female n = 25, collected between 11/17/2006 and 3/15/2019; details are summarized in Supplemental Table 1 and 2). CVID was diagnosed using the ESID criteria (1). Serum samples and diagnostic cell count measurements were obtained after the official CVID diagnosis.

Healthy control (HC, n = 11) sera was obtained from employees of the Laboratory Medical Immunology (LMI) at Erasmus MC (median age 56, range 30-63 years, male n = 4, female n = 7, collected between 09/05/2019 and 10/31/2019 ,summarized in Supplemental Table 1). Additionally, and sera of 18 untreated sarcoidosis patients (SARC) with active lesions (median 47, range 21-80 years, male n = 7, female n = 11, collected between 11/02/2010 and 12/01/2018; details are summarized in Supplemental Table 1) was included.

The study was approved by the medical ethical committee of Erasmus MC (MEC-2013-026, MEC-2021-0652, MEC-2021-0251, MEC-2019-0839).

Categorization of CVID patients

~~Patient files were analyzed to categorize patients according to comorbidities.~~ In total, 35 CVID patients were included. Twenty-three ~~; these were categorized in three groups. If~~ CVID patients suffered from- ~~only suffered from~~ infectious complications, only ~~they were placed in the CVID infections only group~~ (CVID IO~~, n=23~~). Twelve ~~For~~ CVID patients had ~~with~~ additional granulomatous disease (~~n=12,~~ Supplemental Table 2). For serum and cellular analysis shown in Figure 1A-D, 23 CVID IO, seven~~, two groups were formed.~~ CVID patients with non-progressive granulomatous ~~disease at time of sampling were placed in the CVID with granulomatous disease group~~ (CVID+G~~, n=7~~). ~~If samples were collected~~ and seven CVID patients at time of sampling with progressive~~, active~~ granulomatous disease~~, the patient was placed in the CVID with progressive granulomatous disease group~~ (CVID+PG~~, n=7~~) were included. State of granulomatous disease (either non-progressive granulomatous disease or granuloma progression, considered as increased granuloma disease activity) was obtained from patient ~~files (~~reports, radiological examination, long function tests or pathology reports~~)~~. For only two patients (patient 3 and 4) serum samples of paired +G and +PG time points were available for the analysis shown in Figure 1A-D. For the paired analysis shown in Figure 1E, we included additional time points for sIL-2R and the WBC count that were retrieved from routine diagnostic measurements. These measurements were available for patient 3 and 4, and eight of the CVID patients with granulomatous disease (Supplemental Table 2).

Cytokine serum measurements

Serum of CVID patients, sarcoidosis patients and HCs was used for detecting sIL-2R, ACE, sCD14, sCD163 and sCD206. For sCD14 (R&D systems, Human CD14 DuoSet ELISA; DY383-05, serum dilution 1000 times), sCD163 (IQ products, serum dilution 50 times) and sCD206 (Hycult biotech cat#HK381-02, serum dilution 50 times), ELISA was performed according to protocol of the company. ELISAs were performed at research facility of the Laboratory Medical Immunology (LMI) at the Erasmus MC, by an experienced technician.

Serum sIL-2R (by ELISA; Diaclone, Besancon Cedex, France) measurements were also performed at the diagnostic facility of the Laboratory Medical Immunology (LMI) at Erasmus MC under strict quality procedures (ISO15189)). Within the Erasmus MC, sIL-2R levels above 2500 pg/mL are considered elevated, based on serum sIL-2R levels measured in a cohort consisting of 101 healthy donors. These serum sIL-2R measurements were part of regular clinical care and also measured by the same diagnostic facility (LMI) at Erasmus MC using the same validated ELISA system.

For the CVID and sarcoidosis patients and HCs, ACE was determined at the department of Clinical Chemistry of the Erasmus MC, using a commercial ACE kinetic assay kit (Bühlmann Laboratories AG) and was analyzed spectrophotometrically on an automated analyzer (Cobas 8000; Roche Diagnostics).

Absolute cell numbers collection

The white blood cell count (WBC), monocyte, neutrophil, lymphocyte, T- , CD4+ T-, CD8+ T-, B-, and NK cell counts were obtained from routine diagnostic measurements. For 32 CVID patients, serum samples and cellular measurements were collected on the same day. For 14 of the 18 sarcoidosis patients, serum collection and cell count measurements were performed on the same day, and of 4 patients there was a time gap of 3 months between serum sampling and cell count measurements. For 3 CVID patients, cellular measurements were collected not more than three months prior to serum sample collection. For HCs, no matching cellular measurements were available. .

Analysis and statistics

The statistical analyses were either performed in Graphpad Prism V8.0.1. or R version 4.2.3. (2).

Data was considered as nonparametric. For multiple comparison analysis, Kruskal-Wallis with Dunn post-hoc correction was performed in Graphpad. For t-tests, non-parametric distribution was assumed and Mann-Whitney tests were performed. –ROC analysis was performed in Graphpad, where we considered sensitivity the most relevant. Youden index was calculated as follows: (sensitivity + specificity)-1.

In R, the package ggplot2 (version 3.4.0) with Log10 transformed data was used for the PCA analysis, for the heatmap the package ComplexHeatmap (version 2.10.0) was used with Euclidean distance(3).

Supplemental Table S1. Overview included participants, serum markers and cell counts *as shown in Figure 1A-D*

		HC	IO	+G	+PG	SARC	reference value
Group total		11	23	7	7	18	
% Female		63.6	60.9	71.4	85.7	61.1	
Median age at sample collection (range)		56 (30-63)	38 (18-84)	41 (21-70)	48 (14-65)	47 (21-80)	
sIL-2R (ng/ml)	Minimum	1.1	1.0	1.2	12.3	0.6	
	25% Percentile	1.3	1.8	3.6	13.1	2.5	
	Median	1.4	2.9	4.2	14.1	4.0	
	75% Percentile	2.1	4.9	5.3	20.3	5.4	
	Maximum	3.3	16.0	11.2	22.1	9.7	2.5
ACE (U/L)	Minimum	12.5	22.9	30.0	58.0	16.9	12.0
	25% Percentile	32.5	37.8	56.1	64.7	40.3	
	Median	40.0	47.4	57.8	81.3	51.6	
	75% Percentile	51.0	59.5	69.6	142.5	80.3	
	Maximum	63.1	93.2	95.8	239.7	145.9	82.0
sCD163 (ng/ml)	Minimum	467.0	426.7	281.7	1345.0	446.9	
	25% Percentile	540.4	581.8	891.9	1632.0	654.5	
	Median	631.2	866.5	1582.0	2434.0	1205.0	
	75% Percentile	871.1	1227.0	2102.0	6289.0	1772.0	
	Maximum	1311.0	3181.0	2211.0	6833.0	2973.0	
sCD206 (pg/ml)	Minimum	9.2	100.1	121.5	214.6	76.8	
	25% Percentile	121.1	148.1	121.5	218.3	105.6	
	Median	132.5	201.3	156.1	280.6	143.4	
	75% Percentile	228.6	230.5	227.9	331.5	190.3	
	Maximum	316.6	374.2	233.2	730.0	476.4	
sCD14 (ng/ml)	Minimum	877.1	0.1	0.1	0.1	273.1	
	25% Percentile	1149.7	1568.9	1451.4	373.4	1296.1	
	Median	1550.7	1967.8	1895.6	2212.0	2674.9	
	75% Percentile	1654.4	2404.5	2207.8	2994.1	3289.3	
	Maximum	1821.9	3522.8	2716.4	3034.5	4585.6	
T cell (*10^9/L)	Minimum		0.2	0.5	0.5	0.2	
	25% Percentile		0.7	1.0	0.5	0.4	
	Median		1.2	1.7	0.6	0.9	
	75% Percentile		1.7	2.2	1.3	1.4	
	Maximum		2.4	3.4	1.4	2.2	1.9
CD4+T cell (*10^9/L)	Minimum		0.1	0.4	0.1	0.1	0.4

	25% Percentile		0.2	0.4	0.1	0.3	
	Median		0.4	0.9	0.2	0.6	
	75% Percentile		0.5	1.0	0.4	1.1	
	Maximum		1.2	2.0	0.7	1.6	1.3
CD8+ T cell (*10ˆ9/L)	Minimum		0.1	0.1	0.1	0.0	0.2
	25% Percentile		0.4	0.5	0.4	0.1	
	Median		0.8	0.9	0.4	0.1	
	75% Percentile		0.9	1.2	0.9	0.4	
	Maximum		1.4	1.4	0.9	0.7	0.7
CD4/CD8 ratio	Minimum		0.3	0.3	0.2	1.5	2.0
	25% Percentile		0.4	1.0	0.3	1.8	
	Median		0.6	1.1	0.3	2.8	
	75% Percentile		0.8	1.7	1.5	3.4	
	Maximum		2.9	3.6	4.0	9.8	
B cell (*10ˆ9/L)	Minimum		0.0	0.0	0.0	0.0	0.1
	25% Percentile		0.1	0.1	0.0	0.1	
	Median		0.2	0.3	0.1	0.2	
	75% Percentile		0.4	0.4	0.1	0.3	
	Maximum		0.6	0.5	0.2	0.6	0.4
NK cell (*10ˆ9/L)	Minimum		0.1	0.0	0.0	0.1	0.1
	25% Percentile		0.1	0.0	0.0	0.1	
	Median		0.1	0.1	0.1	0.2	
	75% Percentile		0.2	0.2	0.1	0.3	
	Maximum		0.5	0.8	0.2	0.5	0.4
WBC (*10ˆ9/L)	Minimum		2.7	4.4	1.9	3.1	3.5
	25% Percentile		5.2	4.5	2.9	3.8	
	Median		6.8	5.4	3.4	5.0	
	75% Percentile		8.1	6.6	3.8	7.3	
	Maximum		12.1	8.7	4.5	9.7	10.0
sIL-2R/WBC ratio	Minimum		0.2	0.2	3.2	0.1	
	25% Percentile		0.3	0.4	3.6	0.5	
	Median		0.5	0.9	4.4	0.7	
	75% Percentile		0.7	1.0	6.9	1.1	
	Maximum		2.8	2.5	7.0	2.4	

Table S1) IO = CVID with infections only; +G = CVID with non-progressive granulomatous disease; +PG = CVID with progressive granulomatous disease; SARC = sarcoidosis.

Supplemental Table 2. Additional characteristics and analysis groups CVID patients with granulomatous disease

**Patient numbers**	**Sampling groups for Fig.1A-D**	**Included in paired analyses Fig.1E**	**Granuloma location**	**Granuloma biopsy proven**	**Type AI**	**Type malignancy**	**Additional complications**	**IM at time of +G, +PG?**
1	+G	yes	GLILD, liver nodular regenerative hyperplasia suggestive for granulomatous disease	no			lymphadenopathy	no
2	+G	yes	GLILD	yes			lymphadenopathy	no
3	+G & +PG	yes	GLILD, granulomatous thyroiditis	yes	IBD		lymphadenopathy	Yes; at +PG DX, RTX and HCH
4	+G & +PG	yes	Cerebral, GLILD	yes	ITP		no	Yes; at +G CS, at +PG CS, AZA and RTX
5	+PG	yes	GLILD	yes	ITP		no	no
6	+G	yes	GLILD	yes	ITP, AIHA, collagen colitis		lymphadenopathy	Yes; at +G CS and MTX, at +PG DM
7	+PG,	no	abdominal lymph glands, possibly in spleen	yes		Suspected mamma carcinoma (BIRADS 4)	lymphadenopathy and splenomegaly	no

8	+G	yes	GLILD	no	psoriasis	Basal cell carcinoma skin	no	Yes; at +GHCH, at +PG CS and HCH
9	+G	yes	spleen	yes	psoriasis		no	Yes; at +G DX
10	+PG,	no	GLILD	no	ITP, AIHA		lymphadenopathy	Yes; at +PG CS
11	+PG	yes	GLILD	no	psoriasis		no	no
12	+PG	yes	GLILD	yes			no	no

Table S2) +G = CVID with non-progressive granulomatous disease; +PG = CVID with progressive granulomatous disease ; GLILD = Granulomatous Lymphocytic Interstitial Lung Disease; IBD = Inflammatory Bowel Disease; ITP = Immune Thrombocytopenia ; AIHA = Autoimmune Hemolytic Anemia; BIRADS = Breast Imaging-Reporting and Data System; IM = immune modulatory therapy prescribed at time point of sampling or in the three months prior to sampling; CS = corticosteroids, DX = dexamethasone, RTX = rituximab, HCH = hydroxychloroquine, AZA= azathioprine, MTX = methotrexate.

Supplemental Table 3. ROC paramters and Youden's indek sIL-2R/WBC ratio and sIL-2R for groups as shown in Figure 1A-D

		IO vs +G	IO vs +PG	+G vs +PG	SARC vs +G	SARC vs +PG
**sIL-2R/WBC ratio**	Patient numbers	23 vs 7	23 vs 7	7 vs 7	18 vs 7	18 vs 7
	ROC Area	0.6	1.0	1.0	0.5	1.0
	Std. Error	0.1	0.0	0.0	0.1	0.0
	ROC 95% CI	0.4 to 0.9	1 to 1	1 to 1	0.3 to 0.8	1.0 to 1.0
	ROC P value	0.2	<0.001	0.0	0.7	0.0
	cutt-off value	> 837.4	> 2697	> 1735	> 848.2	> 2143
	Sensitivity%	57.1	100.0	100.0	57.1	100.0
	sensitivity (95% CI)	25.1% to 84.18%	64.6% to 100.0%	64.6% to 100.0%	25.1% to 84.2%	64.6% to 100.0%
	Specificity%	82.6	95.7	85.7	72.2	94.4
	specificity (95% CI)	62.9% to 93.0%	79.0% to 99.8%	48.7% to 99.3%	49.1% to 87.5%	74.3% to 99.7%
	Youden's index	0.4	1.0	0.9	0.3	0.9
	Likelihood ratio	3.3	23.0	7.0	2.1	18.0
**sIL-2R**	Patient numbers	23 vs 7	23 vs 7	7 vs 7	18 vs 7	18 vs 7
	ROC Area	0.6	1.0	1.0	0.6	1.0
	Std. Error	0.1	0.0	0.0	0.1	0.0
	ROC 95% CI	0.4 to 0.9	0.9 to 1.0	1.0 to 1.0	0.3 to 0.8	1.0 to 1.0
	ROC P value	0.3	0.0	0.0	0.6	0.0
	cutt-off value	> 3549	> 11694	> 8284	> 4025	> 8499
	sensitivity	85.7	100.0	100.0	71.4	100.0
	sensitivity (95% CI)	48.7% to 99.3%	64.6% to 100.0%	64.6% to 100.0%	35.9% to 94.9%	64.6% to 100.0%
	specificity	56.5	95.7	85.7	50.0	94.4
	specificity (95% CI)	36.8% to 74.4%	79.0% to 99.8%	48.7% to 99.3%	29.0% to 71.0%	74.2% to 99.7%
	Youden's index	0.4	1.0	0.9	0.2	0.9
	Likelihood ratio	2.0	23.0	7.0	1.4	18.0

Table S3) ROC = Receiver-Operator-Curve; CI = Confidence Interval.

References supplementary data

1. Seidel MG, Kindle G, Gathmann B, Quinti I, Buckland M, van Montfrans J, et al. The European Society for Immunodeficiencies (ESID) Registry Working Definitions for the Clinical Diagnosis of Inborn Errors of Immunity. J Allergy Clin Immunol Pract. 2019;7(6):1763-70.
2. Rcore Team. R: A language and environment for statistical computing. R Foundation for Statistical Computing, Vienna, Austria. 2023 [Available from: https://www.R-project.org/.]
3. Gu Z, Eils R, Schlesner M. Complex heatmaps reveal patterns and correlations in multidimensional genomic data. Bioinformatics. 2016;32(18):2847-9.

Supplemental figures

Supplemental Figure S1

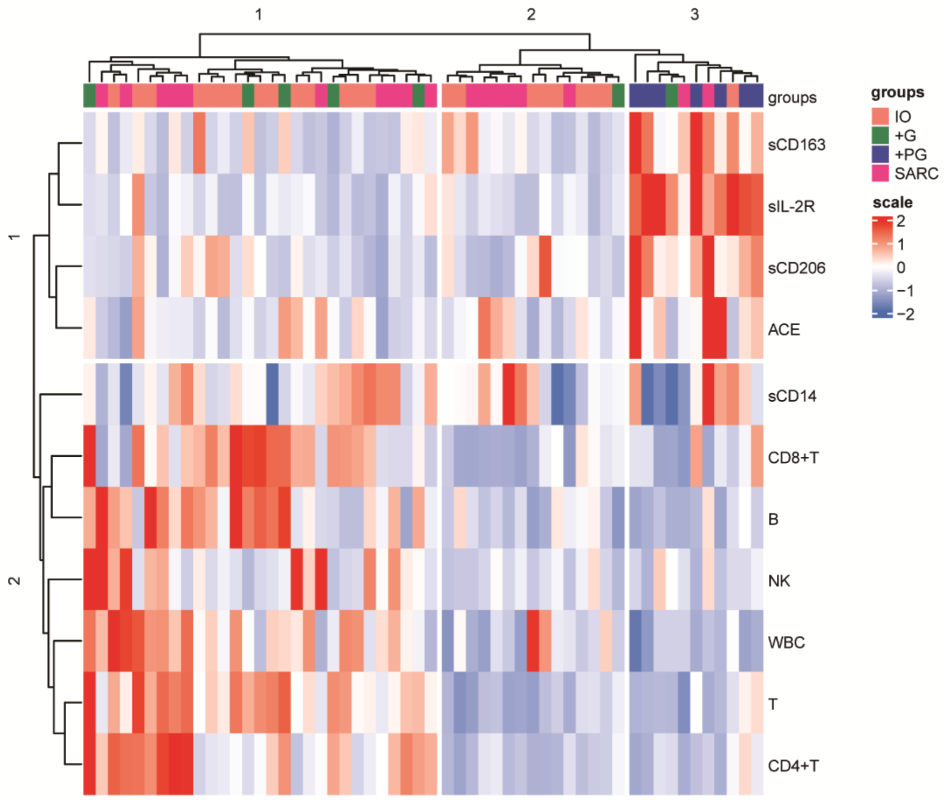

Supplemental Figure S2

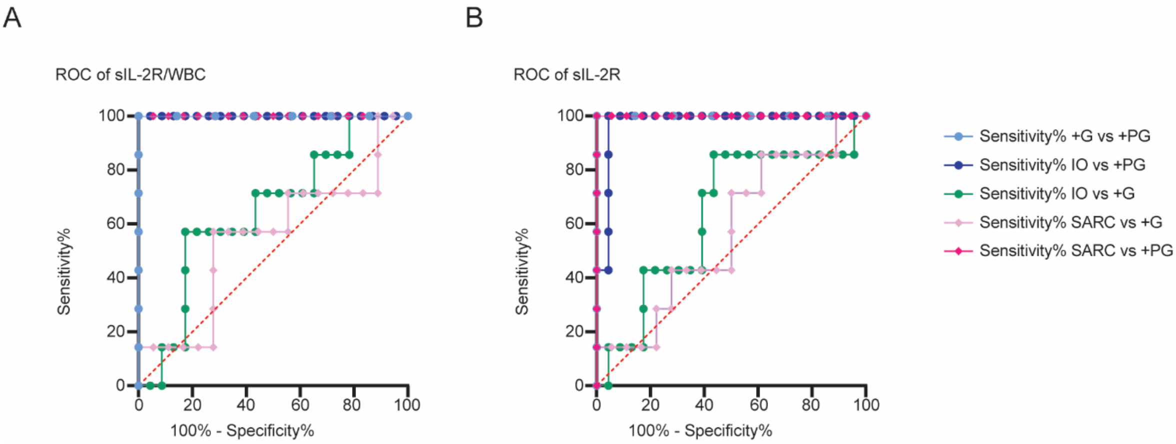

Supplemental Figure S3

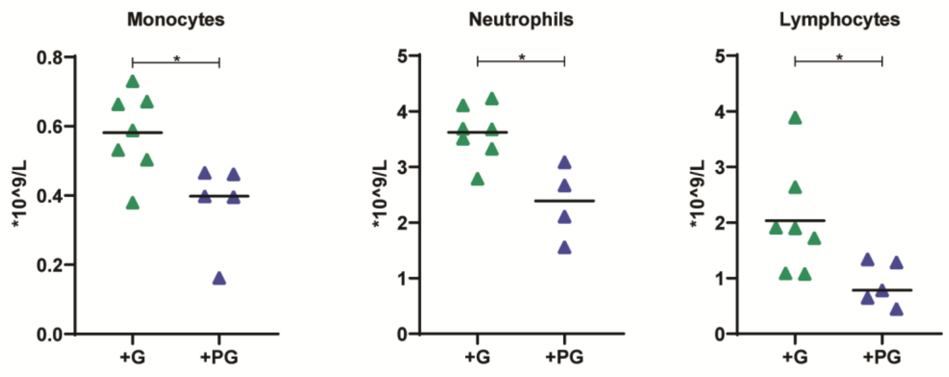

Legends supplemental figures

Legend Supplemental Figure S1

Supp. Figure S1) Heatmap analysis of all soluble serum markers and cell counts, for IO, +G, +PG and SARC, with patient groups as shown in Figure 1A-D, using Euclidean distance. Total number of row and column clusters were preset on 2 and 3.

Legend Supplemental Figure S2

Supp. Figure S2) Receiver-Operator-Curves (ROC) for A) sIL-2R/WBC ratio and B) sIL-2R, for all four patient groups as shown in Figure 1A-D. Sensitivity and specificity is plotted for the following comparisons: IO is set-out against +G, +PG; +G is set-out against +PG and SARC; +PG is also set-out against SARC. Red dashed line indicates a non-discriminatory test. See also Supplemental Table 3.

Legend Supplemental Figure S3

Supp. Figure S3) Monocyte, neutrophil and lymphocyte cell counts of +G and a subset of +PG, with patient groups as shown in figure 1 A-D. Statistical analysis was performed with Mann-Whitney test, *= P <0.05.
